# The Regulation of Induced Depression during a Frustrating Situation: Benefits of Expressive Suppression in Chinese Individuals

**DOI:** 10.1371/journal.pone.0097420

**Published:** 2014-05-14

**Authors:** Jiajin Yuan, Yingying Liu, Nanxiang Ding, Jiemin Yang

**Affiliations:** Key Laboratory of Cognition and Personality (SWU), Ministry of Education, and School of Psychology, Southwest University, Chongqing, China; University of Akron, United States of America

## Abstract

**Background:**

Studies from European-American cultures consistently reported that expressive suppression was associated with worse emotional consequence (e.g. depression) in comparison with acceptance. However, this conclusion may not apply to Chinese, as suppressing emotional displays to maintain relational harmony is culturally valued in East Asian countries. Thus, the present study examined the effects of suppression and acceptance on the depressive mood induced by a frustrating task in a Chinese sample.

**Method:**

Sixty-four subjects were randomly assigned to one of three instructions: suppression, acceptance or no-regulation during a frustrating arithmetic task. The experience of depressive emotion and skin conductance response (SCR) were recorded during pre-frustration baseline, frustration induction and post-frustration recovery phases, respectively.

**Results:**

Compared with the control and acceptance instructions, suppression instruction was associated with decreased depressive experiences and smaller SCR activity during frustration. There were no significant differences between acceptance and control groups in both subjective depression and SCR activity during frustration. Moreover, the suppression group showed a better emotional recovery after the frustrating task, in comparison with the acceptance and control groups. Correlation analyses verified that SCR reactivity was a reliable index of experienced depression during the frustration.

**Conclusions:**

Expressive suppression is effective in reducing depressive experiences and depression-related physiological activity (SCR) when Chinese people are involved. By contrast, the acceptance of depressive emotion in Chinese people does not produce a similar regulation effect. These findings suggest that cultural context should be considered in understanding the emotional consequences of suppression and acceptance strategies.

## Introduction

The ability to regulate emotions is important for social adjustment and well-being. Amongst various emotion regulation strategies, expressive suppression is defined as the conscious, effortful attempts to hide emotional responses and constrain emotion-expressive behaviors [1], [2]. For instance, when a person feels angry, he/she tries to dampen anger experience by hiding this emotion and pretending to be calm [1], [2]. A number of researches showed that suppression was associated with elevated levels of physiological arousal and negative affective consequences [3]–[8]. Moreover, studies on thought and pain suppression also suggested that suppression might be associated with a paradoxical persistence of the unwanted thought and pain [9], [10].

By contrast, acceptance is an effortless, exposure-based strategy that is defined as "the aware embracing of emotional events and the active experiencing of their emotional consequences, without attempts to change the frequency, form or impacts of the emotional events [11]. Acceptance is considered an important component of Acceptance and Commitment Therapy [11], [12], and the application of the acceptance strategy is associated with a line of positive outcomes, including lower levels of negative affect [13]–[15], decreased anxious or depressive symptoms [16]–[20], and better physical/social functioning [21]. Recently, Shallcross showed that accepting negative emotions might protect individuals from experiencing negative affect and developing depressive symptoms [22].

Researchers have proposed acceptance and suppression as two ends of the experiential avoidance continuum, with higher levels of experiential avoidance reflecting higher emotional suppression, that is, less willingness to experience negative emotions [15], [23]–[25]. Similarly, “high acceptance” is considered to be synonymous with “low experiential avoidance” [15], [26]. Driven by this theoretic construct, many studies compared suppression and acceptance strategies in the behavioral and physiological effects of emotion regulation [5], [13], [14].

For example, Hayes and colleagues showed that participants who received an acceptance-oriented rationale prior to a cold-pressor task displayed greater pain tolerance than those who received suppression-oriented or placebo rationales [27]. Moreover, Eifert and Heffner [28] compared the effects of acceptance and suppression strategies on the avoidance of panic-relevant interoceptive stimulation, which was elicited by carbon dioxide enriched air. The results showed that the acceptance group reported less intense fear, fewer catastrophic thoughts and greater willingness to return for another experimental session than the suppression group [28]. Levitt and colleagues [29] replicated this study with a sample of individuals diagnosed with pain disorder. They found similar effects of acceptance in this clinical sample, including decreased subjective distress and increased willingness to undergo another symptom provocation [29]. In addition, Campbell and colleagues [14] investigated perceived acceptance and suppression of negative emotion in participants with anxiety and mood disorder in response to a distressing film. The results showed that high levels of suppression were associated with negative emotion during the film and post-film recovery period [14]. Campbell [13] also found that the acceptance group displayed less negative affect during a post-film recovery period. Furthermore, the suppression group showed increased heart rate, whereas the acceptance group displayed decreased heart rate in response to the film [13]. Recently, Hofmann and colleagues [5]directly compared the behavioral and physiological indexes of regulation using expressive suppression, acceptance and cognitive reappraisal during an impromptu speech task. The results suggested that the suppression group showed a greater increase in heart rate from baseline than the acceptance and reappraisal group [5].

These studies consistently observed that suppression was associated with negative outcomes, whereas acceptance was linked to beneficial consequences. However, most of these studies were conducted in European- American samples that are characterized by individualistic cultural values encouraging free emotional expressions [30], [31]. By contrast, East Asian cultures are characterized by collectivistic cultural norms, which encourage the suppression of emotional displays, in order to avoid hurting others and maintain relational harmony [30]–[34]. In fact, a growing number of studies have indicated that the emotional consequence of suppression is cultural- specific: suppression is related to less negative emotional experience, better social interactions and more favorable physiological response in individuals with East Asian cultural values [35]–[37]. For example, Butter and colleagues [35] observed that greater suppression was associated with enhanced negative emotions, increased hostile behaviors or negative interpersonal perceptions in European Americans. By contrast, these adverse outcomes were reduced or reversed in Asian Americans who held Asian values [35]. In addition, Mauss and colleagues [36] showed that emotional restraint led to smaller anger experience and reduced anger-expressive behaviors, as well as smaller cardiovascular activity during anger provocation among Asian Americans, but not among European Americans. Consistent with these findings, Soto [37] observed that the relationship between suppression and health depended on cultural context. Specifically, suppression was associated with adverse psychological functioning for European Americans, but not for Chinese participants [37]. Since many studies suggest that suppression is an adaptive and effective emotion regulation strategy in East Asian cultures, it seems unlikely that the suppression is less effective than acceptance in regulating negative emotions in Chinese. Instead, suppression might be as similarly effective as, or even more effective than, acceptance in regulating negative emotion in Chinese subjects.

A number of evidences suggest that individuals with sub-threshold depression had an increased risk of major depression and other adverse outcomes [38]–[40]. Though previous studies examined behavioral and physiological consequences of negative emotion regulation in multiple emotion categories, such as anxiety [5], anger [41], sadness [42] or composite negative emotion [13], [14]; few studies have investigated the regulation of situational depressive mood by comparing suppression and acceptance strategies. Thus, it is practically important to study the efficacy of regulating nonclinical state depression by acceptance and suppression.

Most previous studies used emotional pictures or film clips to induce negative emotion during emotion regulation [13], [14], [43], [44]. These pictures and films depicted emotionally salient scenarios, like surgical operations [45], persecutions [13], [14], disasters and threats [46]–[48], to elicit prominent emotional reactions in observers through emotional perception or contagion [49], [50]. However, the emotional event that evokes intense emotions often has close personal relevance in life settings. This is often the case, in our life experiences, that self-experienced bereavement induces much stronger sadness than the perception of bereavement scenarios. This argument was supported by a couple of studies which observed higher ventromedial prefrontal reactions to emotional pictures with personal relevance [51], and greater anger perception from faces with direct gaze to observers [52]. Thus, to induce depressive emotion with high ecological validity, it is necessary to use a task that entails self-involvement during emotion induction.

Therefore, we adopted a frustrating arithmetic task, which required subjects to rapidly count the number of triangles embedded in complex graphics to induce depressive emotion. A feedback was provided about the performance in each trial, and the valence of the feedback was determined by a computer program that displayed negative feedback 18 out of 20 trials. Thus, this task constituted a frustrating situation, and many studies showed that the frustrating task with negative feedbacks was effective in inducing negative, depressive emotional state [53]–[56]. For instance, Goodwin and Williams reported in an early study that experimental manipulations of success and failure induced depressive and negative affect [56]. In addition, it was reported that the negative feedback was associated with depressive affects, with enhanced depression symptoms predicting hypersensitivity to negative feedback [57]–[59].

In sum, using a frustrating arithmetic task, the objective of the present study was to examine the effects of suppression and acceptance on state depression responses to frustration in local Chinese subjects. The subjective emotion experience and physiological activity (SCR) were measured in three phases: the rest phase, the frustrating task phase, and the recovery phase. Skin conductance response has been deemed as excellent index of emotional arousal [60]. Prior studies indicated that the SCR has advantage over other autonomic measures (e.g. heart rate) in reflecting emotional arousal, because SCR is under strict control of the sympathetic branch of the nervous system [61]. Moreover, SCRs had been accepted as reliable measures of autonomic expressions of emotions [61]. Because East Asian culture is associated with favorable outcomes during emotion-expressive suppression, we predicted that suppression might be as similarly effective as, or even more effective than, acceptance in regulating subjective experience of state depression and physiological arousal (SCR) in Chinese subjects.

## Methods

### 2.1. Subjects

**Figure 1:**
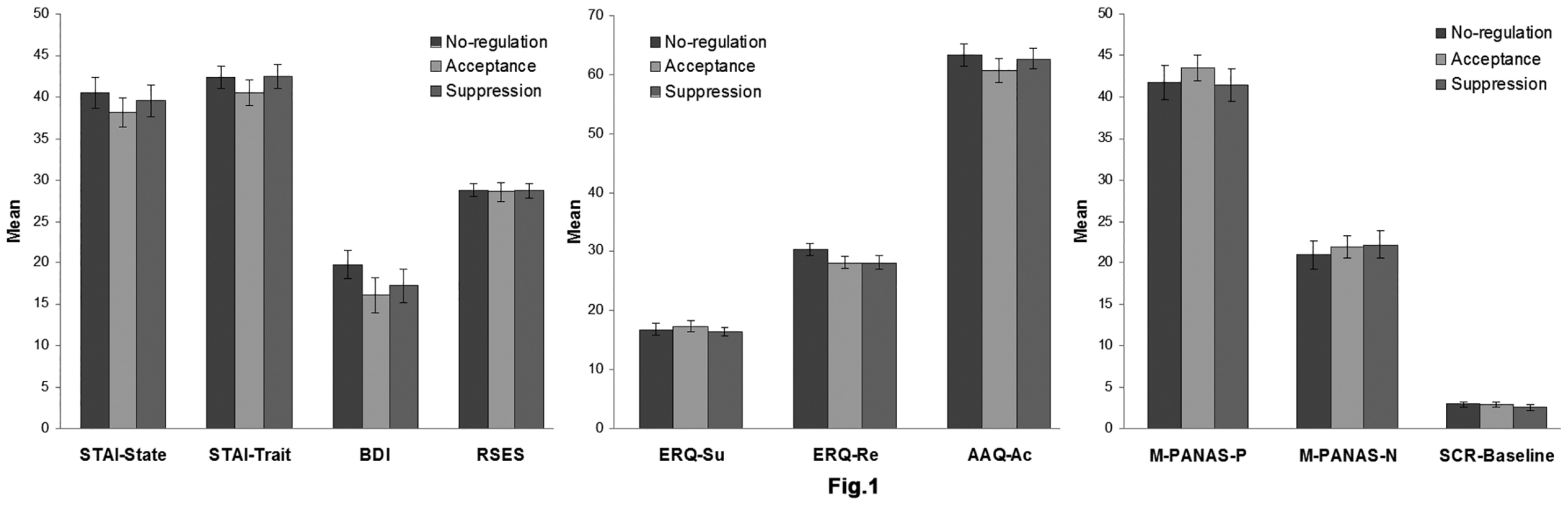
Means of the emotion-related and baseline measurements for the three groups. Error bar represents standard error.

As paid volunteers, 64 undergraduate students aged 18 to 24(*M* = 20.52, *SD* = 1.34) from South University in China participated in the experiment. They were randomly assigned to three different conditions: no-regulation (N = 20), acceptance (N = 21) and suppression (N = 23). All the subjects reported no history of affective disorder and were free of any psychiatric medication. There were no significant group differences in pre-experiment emotional states, as indicated by the similar scores in Spielberg State Anxiety Scale [62] *F*(2,61) = .394, *p* = .676, Spielberg Trait Anxiety Scale [62] *F*(2,61) = .561, *p* = .574, and Beck Depression Inventory-II [63] *F*(2,61) = .870, *p* = .424. Also, the three groups were controlled in the measures of Rosenberg Self-Esteem Scale [64] *F*(2,61) = .016, *p* = .985 (see Fig.1 and Table.1), as self-esteem was negatively correlated with depressive responses during stressful situations [65]. In addition, there were no significant group differences in the habitual use of cognitive reappraisal *F*(2,61) = 1.329, *p* = .272, expressive suppression *F*(2,61) = .265, *p* = .768, and acceptance *F*(2,61) = .449, *p* = .640 (see Fig.1 and Table.1). As reappraisal is an extensively used strategy during emotion regulation, we also measured and equated the habitual use of reappraisal across the three groups. The subjects were right-hand and had normal or corrected to normal vision. The study was approved by the human subjects review board of Southwest University in China. The experimental procedure was in accordance with the ethical principles of the 1964 Declaration of Helsinki (World Medical Organization, 1996). Each subject signed an informed consent form before the experiment.

**Table 1:** The emotion-related and baseline measurements.

	Control(20)	Accept(21)	Suppress(23)
Gender	9F/11M	11F/10M	13F/10M
STAI-State	40.50 (8.29)	38.14 (7.79)	39.52 (9.41)
STAI-Trait	42.35 (6.05)	40.48 (7.73)	42.43 (6.91)
BDI	19.75 (7.60)	16.10 (9.66)	17.22 (9.67)
RSES	28.80 (3.45)	28.57 (4.98)	28.70 (3.87)
ERQ-SU	16.80 (4.23)	17.29 (4.30)	16.39 (3.71)
ERQ-RE	30.30 (4.57)	28.10 (4.78)	28.09 (5.58)
AAQ-AC	63.25 (8.42)	60.76 (9.50)	62.65 (8.54)
M-PANAS-P	41.75 (8.97)	43.48 (7.16)	41.48 (9.44)
M-PANAS-N	20.95 (7.84)	23.71 (6.99)	24.43 (8.96)
SCR-Baseline	2.98 (1.62)	2.92 (1.57)	2.58 (1.37)

Note–Data are mean (standard deviation) values. STAI  =  Spielberger State Trait Anxiety Inventory; BDI  = Beck Depression Inventory-II; RSES  =  Rosenberg Self-Esteem Scale; ERQ  =  Emotion Regulation Questionnaire; AAQ  =  Acceptance and Action Questionnaire; M-PANAS  =  Modified Positive Affect Negative Affect Scale.

### 2.2 The behavioral procedure and the frustrating arithmetic task

Subjects were seated in an acoustically isolated room at approximately 150 cm from a computer screen. The task consisted of 20 trials. Each trial started with a small white fixation cross on the black computer screen for 800 ms to remind subjects of the following task. Then, a complex geometric figure was presented for 39 seconds, which is the mean time of another 40 subjects used to identify the number of triangles in a pre-test. Subjects were instructed to count the number of the triangles from the complex figure within 39 s time limit. Then, an answer screen was presented, and subjects needed to enter their answers as soon as possible. Once inputting the answer, the feedback was provided for 300 ms. The feedback was manipulated to be negative in 18 trials (“wrong”) and to be positive in 2 trials (“correct”). Subjects would see a general feedback for 30000 ms at the end of the experiment, which reminded subjects that their overall accuracy was 10%. The whole experiment was divided into three phases: rest (T1), task (T2) and recovery (T3) phases. The affective state measured by the modified version of Positive Affect and Negative Affect Scale (PANAS) and skin conductance responses (SCR) were recorded during each phase. The whole experiment lasted for about 30 minutes in a quiet room where temperature was set to 26°C

#### 2.2.1: Rest phase

This was the beginning phase of the experiment, and in this phase subjects were instructed to do nothing but to relax quietly for 3minutes with eyes closed. After the 3 mins rest, they completed the modified PANAS.

#### 2.2.2: The task phase and Emotion regulation instructions

In the task phase, we employed a frustrating arithmetic task to induce depressive emotion. Prior to the task, subjects received suppression, acceptance or no-regulation instructions, according to their group assignment.

The subjects in the suppression group received instructions: “your task is to count the number of triangles embedded in the complex figure within 39 seconds, which is the mean time used by another 40 students to finish this job. After the offset of the complex figure, there was an answer screen where you need to enter your answer as soon as possible. Once input the answer, you will be informed of the correctness of your counting (“wrong” or “correct”).The task is a bit difficult and therefore you may sometimes experience frustration. If this happens, please try to control your negative emotional expression and do not let them show, such that a person watching your face would not know what you feel [2], [3], [5], [66].”

The acceptance group received the same instruction for the triangle-counting task. Moreover, they received a specific acceptance instruction as follows: “The task is a bit difficult and therefore you may sometimes experience frustration. If this happens, please try to accept and experience your negative emotion naturally and not to change or control them in any way. Let your emotion run naturally, and think of them as natural phenomena, just like a cloud passing in the sky. Please allow yourself to stay harmoniously with your negative emotions [13], [14].”

The subjects in the control group received no additional emotional regulation instruction except for the same triangle-counting instruction.

All the subjects practiced four trials to familiarize themselves with the task. Then, subjects performed the frustrating task for approximately 30 minutes. Afterwards, they rated the extent to which they suppress/accept negative emotion during the task phase. Finally, subjects were asked to complete the modified PANAS.

#### 2.3.3 Recovery phase

Immediately after completing the task, subjects were asked to rest for 3minutes with eyes closed during the recovery phase. After the 3 mins recovery, they completed the modified PANAS. Lastly, subjects completed the Emotion Regulation Questionnaire (ERQ) [67] and the Acceptance and Action Questionnaire (AAQ) [68]. The ERQ a 10 item self-report measure that assesses chronic use of cognitive reappraisal and expressive suppression, with each item scoring from 1 (not at all true) to 7 (very true). The 16-item version of AAQ (7-point scale) is widely adopted as self-report measure to assess the dimension of acceptance versus experiential avoidance.

### 2.4. Dependent variables

#### 2.4.1 The modified version of PANAS

Positive and Negative Affect Scales [69] is a 20-item self-report measure with 10 items measuring positive affect and 10 items measuring negative affect. Items are rated on a 5-point Likert scale, ranging from 1 (“very slightly or not at all”) to 5 (“extremely”). Psychometric evaluation of the PANAS indicates that this scale has satisfactory reliability and validity [69], [70].

To detect affective changes more sensitively, the present study administered the PANAS on 7-point Likert Scale, ranging from 1 (“very slightly or not at all”) to 7 (“extremely”). Because frustration is characterized by depressive mood [57], [59], we directly measured levels of depressive mood by adding an item of “depressive” to the PANAS measurement.

#### 2.4.2. Physiological measurement

Skin conductance response (SCR) was taken with the NeXus-10 Mark II system (Mind Media B.V. Netherlands), which uses the BioTrace+ application software for data analysis and presentation. The SCR was measured using the SC electrodes, which were attached to the medial phalanx of the index and middle fingers on subject's left hand. The signal was sampled with a frequency of 32 Hz. Subjects washed their hands with soap and water before the electrodes were attached. During the collection of physiological data, the onset and offset of each phase of interest were defined using an event marker. Average values of SCR were computed for each phase of interest (e.g., rest, task, recovery) using the BioTrace+ software.

## Results

### 3.1 The validity of depression induction

As has been established, the emotional symptoms of depression were composed of feelings of sadness, upset, irritability, enthusiasm/interest shortage, worthlessness, hopelessness, guilt and self-devaluation [63], [71]–[75]. Therefore, if our task effectively induced a depressive emotional state, the task phase should be not only associated with enhanced depressive report but also with enhanced negative emotion measures such as upset, irritable, ashamed, guilty, distressed, enthusiasm shortage and worthlessness.

**Figure 2:**
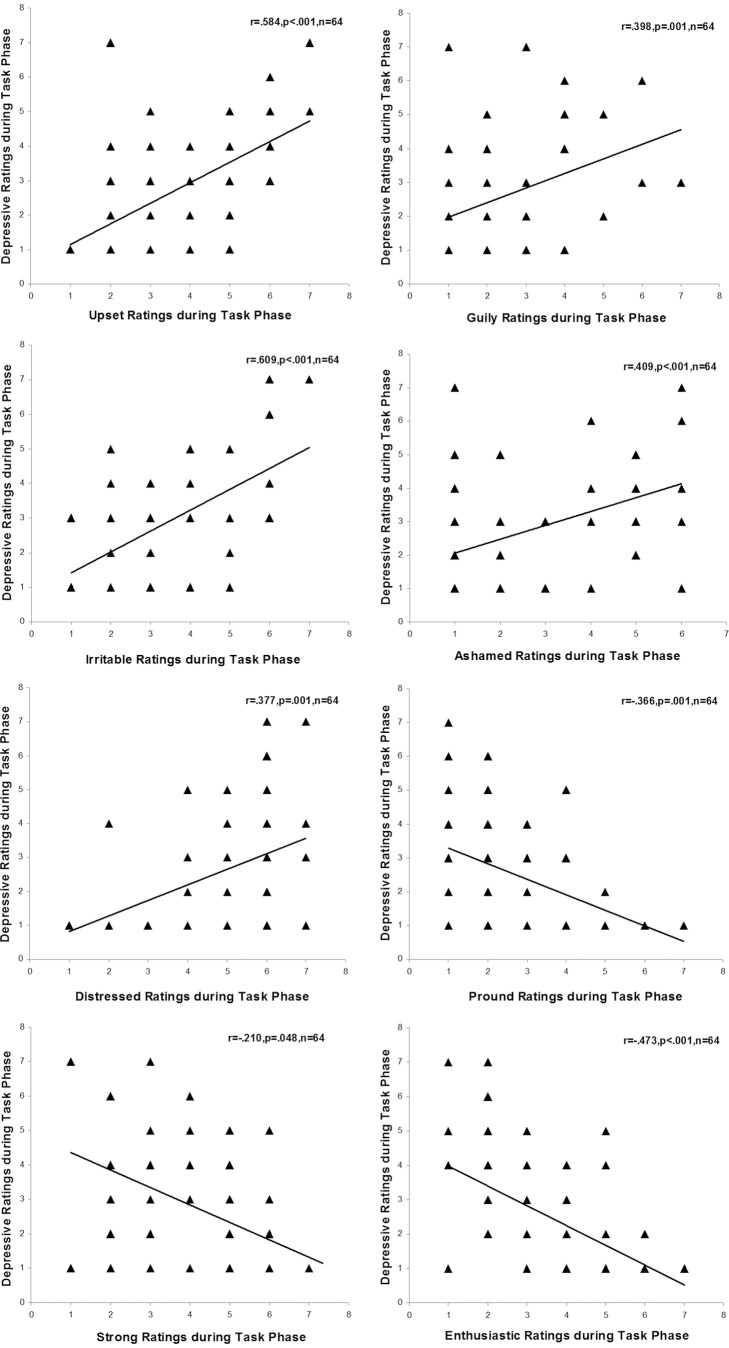
Scatterplots for the correlation between state depression and other eight emotions that significantly changed during the frustrating task.

Thus, we examined the validity of depression induction by the following ways. Firstly, we examined the emotions that significantly changed during the task. The Paired-Samples T Test was conducted to compare the ratings of each emotion (measured by the modified PANAS) between the rest phase and the task phase in control subjects. The Paired-Samples T Test was conducted in the control group, because this group was free of regulation instructions. The results showed that six negative emotions were significantly induced by the task, including the items of depressive *t*(19) = −4.344 *p*<.001, upset *t*(19) = −6.206 *p*<.001, irritable *t*(19) = −3.380 *p* = .003, guilty *t*(19) = −3.003 *p* = .002, ashamed *t*(19) = −3.214 *p* = .005, distressed *t*(19) = −2.979 *p* = .008. In addition, three positive emotions were significantly reduced during the task phase, including the items of proud *t*(19)  = 4.188 *p*<.001, strong *t*(19)  = 2.545 *p* = .020, enthusiastic *t*(19)  = 3.526 *p* = .002. Then, the Pearson correlations were conducted to examine the relationship between the depression report and the eight emotions mentioned above, respectively. The results showed that there was a significantly positive correlation between the depressive ratings and every other five negative emotions, *r*_min_ = .377 *p*_max_ = .001 n = 64 (Fig.2). Furthermore, there was a significantly negative correlation between the depressive ratings and every other three positive emotions, *r*_min_ = −.210 *p*_max_ = .048 n = 64 (Fig.2). Based on these results, subjects felt upset, irritable, guilty, ashamed, and distressed during the task. Meanwhile, they felt less proud, less strong, and less enthusiastic. These results verified that the current frustration task effectively induced depressive emotional state.

### 3.2 Manipulation checks

The first manipulation check was to examine whether the subjects correctly understood and adopted the emotion regulation strategies. After reading the emotion regulation instructions, subjects were asked to describe them according to their understandings, which would be corrected timely if wrong. In addition, at the end of practice training, subjects were instructed to rate how proficiently they used the regulation instructions by a 6-point scale (1: not at all; 6: extremely). If the ratings were under four, the subjects would be instructed to practice again. All subjects met the first manipulation check.

The second manipulation check aimed to examine whether subjects complied with the emotion regulation instructions during the task. Subjects were asked to rate the extent to which they accept (acceptance group) or suppress (suppression group) their emotions by a 6-point scale (1: not at all; 6: extremely) immediately after the task. The analysis of the instruction conformation ratings showed that the acceptance strategy was successfully used in the acceptance group (*M±S.E.*:4.38±0.16),and the suppression strategy was successfully used in the suppression group (*M±S.E.*:4.17±0.18). The scores were significantly higher than the midpoint of the rating scale (i.e. 3) in both the acceptance group (*t* (20) = 8.55, *p*<0.001) and suppression group (*t* (22) = 6.35, *p*<0.001). The ratings were not significantly different between the acceptance and suppression groups (*t* (42) = 0.84, *p* = 0.41).

### 3.3 Group comparability

Sixty-four subjects were randomly assigned to one of the three groups (control, acceptance, suppression). The three groups were comparable in their baseline levels of the positive and negative affect ratings, and the SCR during the rest phase. There were no significant group differences on any of the baseline measurements, *F_max_* = 0.461, *P_min_* = 0.633 (see Fig.1 and Table1). This suggested that the three groups of subjects were similar in the pre-experiment baseline emotional states.

### 3.4 Effects of emotion regulation on depressive ratings

**Figure 3:**
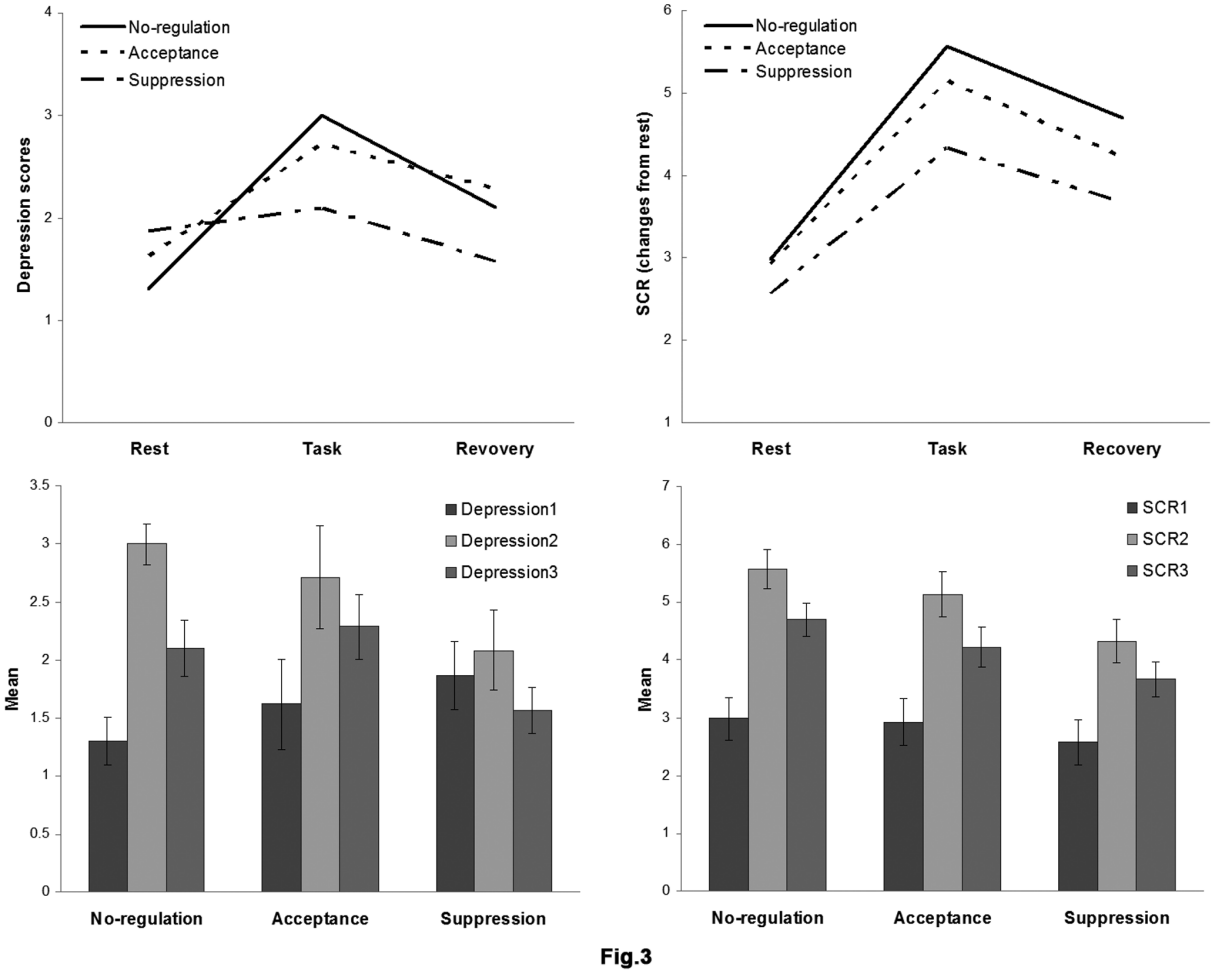
Depressive scores and SCR values between the emotion regulation strategies in the rest, task and recovery phases. Error bar represents standard error.

In order to explore the impact of acceptance and suppression on the depression induced by the frustrating arithmetic task, we conducted a 3 (phase)×3 (group) repeated measures ANOVA on depressive ratings, with phase (rest, task, recovery) as a within-subjects variable, and grouping as a between subjects variable (control, acceptance, and suppression). The results revealed a significant main effect of phase, *F* (2,122) = 17.542 *p*<.001 *η^2^_p_* = .223, and a significant phase by group interaction effect, *F* (4, 122) = 3.803 *p* = .006 *η^2^_p_* = .111 (see Table.2 and Fig.3). To break down the interaction, we tested the effect of phase in each of the three regulation groups. There was a significant phase effect in the control group (*F* (2, 38) = 12.965, *p*<.001, *η^2^_p_* = .406). A post-hoc test with Bonferroni correction revealed increased depressive ratings from the rest phase to task phase (p<.001), and a significant reduction from the task phase to recovery phase (p = .028). In addition, there was a significant phase effect in the acceptance group, (*F* (2.40) = 5.063, *p* = .011, *η^2^_p_* = .011). The Post hoc test with Bonferroni correction revealed increased depressive ratings from the rest phase to task phase (p = .044), while the reduction of depressive ratings from the task to recovery phases were statistically non-significant (p = .166). The suppression group also showed a significant phase effect in depressive ratings (F (2, 44) = 3.34, p = .045, η2p = .132). The post-hoc test with Bonferroni correction revealed no significant increase in depressive ratings from the rest phase to task phase (p = .656), but a significant reduction in depressive ratings from the task phase to recovery phase (p = .040; see Table 2 and Fig.3).

**Table 2:** Depressive scores and SCR measures during rest, task, and recovery phase.

		Control(20)	Accept(21)	Suppress(23)
Depressive	Rest	1.30 (0.92)	1.62 (0.80)	1.64 (1.04)
	Task	3.00 (1.75)	2.71 (2.05)	2.09 (1.31)
	Recovery	2.10 (1.29)	2.29 (1.59)	1.57 (0.95)
SCR	Rest	2.98 (1.62)	2.92 (1.57)	2.58 (1.37)
	Task	5.57 (1.80)	5.14 (1.81)	4.32 (1.68)
	Recovery	4.70 (1.75)	4.22 (1.72)	3.67 (1.47)

Note–Date are mean (standard deviation) values. SCR recorded in mS (micro-siemens).

To further explore if the size of rest-to-task increases and the task-to-recovery decreases differ significantly across groups, we computed two new variables for each of the three groups: D1 =  T2-T1 (rest-to-task increase), and D2 = T2-T3 (task-to-recovery decrease) and then tested the grouping effect in the two variables, respectively. There was a significant grouping effect in D1 (*F* (2, 61) = 4.468, *p* = .015). The post hoc test with Bonferroni correction revealed a significantly smaller D1 in suppression (*M±S.E:*0.22±0.22) compared to the control (*M±S.E:1.70*±0.39) groups (*t*(41) = 3.427, p = .013), suggesting that the suppression group showed smaller increases in depressive ratings compared to the control group. However, the depressive ratings were not significantly different between the control and acceptance (*M±S.E:1.10*±0.44) groups (p = .73). For D2, there were no significant group differences (*F* (2, 61) = 0.898, *p* = 0.413). To further assess whether the quality of emotion recovery differs across groups, we computed recovery-rest differences (defined as D3 = T3-T1) in depressive ratings and tested whether D3 differs across groups. There was a significant grouping effect in D3 (*F* (2, 61) = 5.378, p = .007), which was smaller in suppression (M±S.E:−0.30±0.18) compared to the control (M±S.E: 0.80±0.27; t(41) = 3.484, p = .013) and acceptance (M±S.E:0.67±0.0.33) groups (t(42) = 2.616, p = 0.032). This suggests that suppression produced best emotion recovery to the baseline compared to other instructions. There was no significant difference between the control and acceptance group (p = 0.10).

### 3.5 Effects of emotion regulation on SCR

In terms of SCR, the 3×3 repeated measures ANOVA showed a significant main effect of phase, *F* (2, 122) = 212.963 *p*<.001 *η^2^_p_* = .78 and a significant Phase by Group interaction, *F* (4, 122) = 2.929 *p* = .024 *η^2^_p_* = .09 (see Table.2 and Fig.3). To break down the interaction, we tested the phase effect in each of the three groups. There was a significant phase effect in the control group (*F* (2, 38) = 147.785, *p*<.001, *η^2^_p_* = .886). The post-hoc test with bonferroni correction revealed elevated SCR from the rest to task phases (*p*<.001), and a significant reduction from the task to recovery phases (*p*<.001). Similarly, there was a phase effect in the acceptance group, (*F* (2,40) = 74.115, *p*<.001, *η^2^_p_* = .787), with SCR levels increased from the rest to task phases (*p*<.001), and decreased from the task to recovery phases (*p*<.001, bonferroni corrected). The suppression group also showed a significant phase effect in SCR (*F* (2, 44) = 36.606, *p* = <.001, *η^2^_p_* = .625). A post-hoc test with bonferroni correction revealed a significant increase in SCR from the rest phase to task phase (*p*<.001), and a significant reduction in SCR from the task phase to recovery phase (*p*<.001; see Table 2 and Fig.3).

To explore if the rest-to-task increases, the task-to-recovery decreases, and the post-task emotional recovery differ across groups, we computed three variables in each group: S1 =  T2-T1 (rest-to-task increase), and S2 = T2-T3 (task-to-recovery decrease) and S3 =  T3-T1 (rest-to-recovery increase). Then, we tested the grouping effect in the three variables, respectively. There was a significant grouping effect in S1 (*F* (2, 61) = 3.549, *p* = 0.035), which was significantly smaller in suppression (M±S.E:1.75±0.26) compared to the control (M±S.E: 2.59±0.17) groups (t (41) = 2.652, p = .031), suggesting that the suppression group showed smaller increases in SCR compared to the control group. However, the SCR was not significantly different between the control and acceptance (M±S.E: 2.22±0.23) groups (p = .790). There were no significant group differences for S2 (F (2, 61) = 2.598, p = 0.083) and S3 (F (2, 61) = 2.275, p = .111).

### 3.6 Correlation analyses

**Figure 4:**
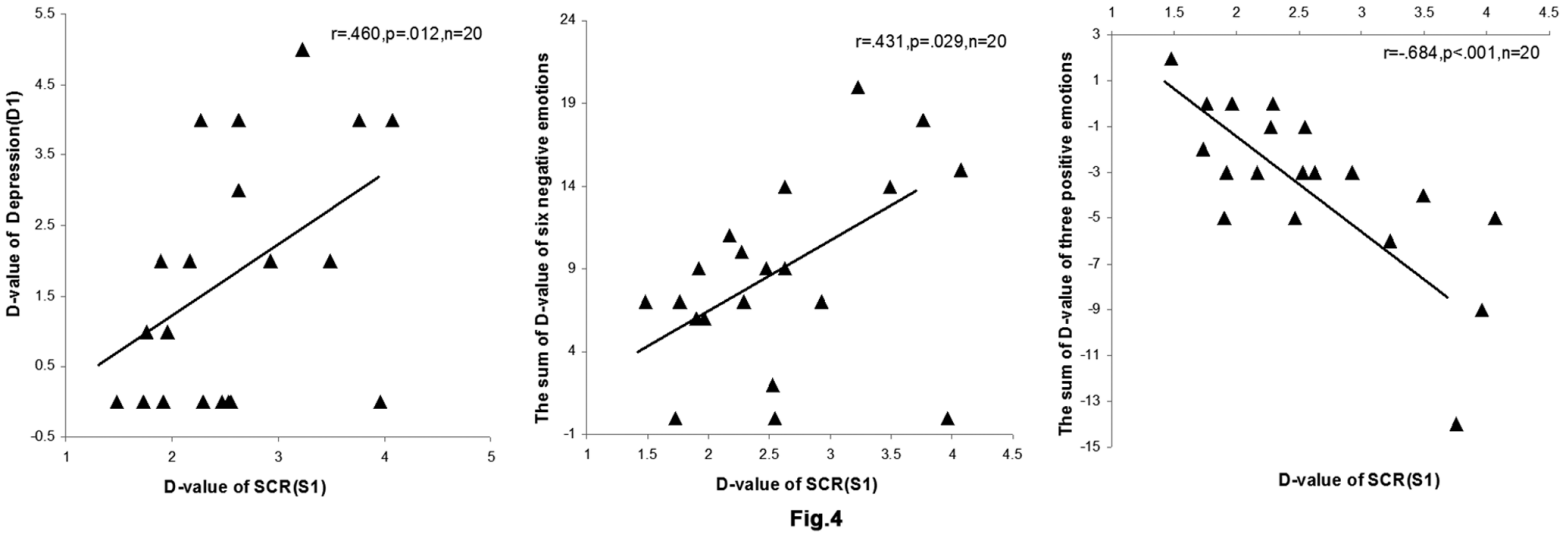
Scatterplots for the three correlations in the control group: between SCR (S1) and the state depression (D1); between SCR (S1) and the sum of the task-rest difference scores for the six negative emotion measures (depressive, upset, irritable, guilty, ashamed, and distressed) that were induced by the frustration task; and between the SCR (S1) and the sum of the task-rest difference scores for the three positive emotion measures (proud, strong, and enthusiastic) that significantly decreased during the frustration task.

To verify whether SCR is a valid index of depressive emotion, the spearman correlation was performed between the depressive emotion (D1) and SCR (S1) increases from rest to task phases in the control group. The correlation was conducted in the control group because this group was free of regulation instructions. The results showed that SCR was correlated significantly and positively with depressive experience (*r* = .460, *p* = .021, *n* = 20; see Fig.4). To clarify whether this correlation was specific to depressive emotion, a similar correlation was conducted between the SCR (S1) and the sum of the task-rest different scores for the five negative emotion measures (scared, hostile, nervous, afraid, and jittery) that were not induced by the frustration task. Consistent with our prediction, this correlation did not reach significance (*r* = .284, *p* = .113, *n* = 20). Because depressive mood was characterized by feelings of ashamed, upset, irritable, guilty and distressed that were significantly increased during the frustration task, we also conducted the Spearman correlation between the SCR (S1) and the sum of the task-rest different scores for these six negative emotion measures (depressive, upset, irritable, guilty, ashamed, and distressed). The result showed a significant positive correlation between the two variables (*r* = .431, *p* = .029, *n* = 20; see Fig.4). In addition, previous studies indicated that the loss of pleasurable engagement is an important feature of depression [76]–[80], such as enthusiasm shortage and worthlessness [63], [72], [74]. Consistent with the findings, Subjects felt less proud, less strong, and less enthusiastic during the frustration task. Therefore, we conducted the Spearman correlation between the SCR (S1) and the sum of the task-rest difference scores for the three positive emotion measures (proud, strong, and enthusiastic) that decreased during the frustration task. The results revealed a significant negative correlation (*r* = −.684, *p*<.001, *n* = 20; see Fig.4), suggesting that the SCR levels were enhanced as a function of decreasing positive emotions. Therefore, SCR activity is a valid index of the depressive emotion in the present study.

## Discussion

Many studies in western cultures suggest that suppressing the expression of negative emotion was maladaptive, because expressive suppression enhanced subjective negative affect and physiological arousal [3]–[5], [7], [8], [66]. For instance, the early studies by Gross and colleagues observed that expressive suppression did not decrease the subjective experience of negative emotions and physiology arousal [3], [4]. In addition, there was evidence showing that emotional suppression led to unfavorable psychological consequences [81], [82], such as more intrusions of negative experiences and more obsessive-compulsive problems [81], [83], [84]. On the other hand, it has been reported that acceptance is an adaptive strategy that produces a line of positive psychophysiological outcomes [13]–[20], [22]. For instance, it was reported that acceptance instruction decreased heart-rate during distressful films, and reduced negative affects during post-film recovery periods [13]. Also, it was reported that habitual acceptance was associated with decreased negative affects and less depressive symptoms during stressful situations [22].

Contrary to the previous findings, the present study observed that suppression was effective in reducing the depressive experience and its physiological arousal (SCR), while acceptance did not yield positive effects at both subjective and physiological levels. Specifically, suppression group showed no significant increase in depressive ratings from rest to task phases, but a significant decrease from task to recovery phases. By contrast, acceptance group showed a significant increase from rest to task phases, but no significant decrease from task to recovery phases. These findings were replicated by our analyses of the task-rest differences in depressive feelings and SCR activity, which demonstrated smaller rest-to-task increases in both indexes during suppression in comparison with the control groups. The efficacy of expressive suppression in regulating induced depression was also supported by our analyses of the rest-to-recovery differences in depressive experience (D3), which showed a better return to the pre-experiment baseline in the suppression group. By contrast, the acceptance group did not show any significant differences in all these measures compared to the control group. These results suggest that the suppression is more effective in regulating the subjective experience and physiological arousal (SCR) associated with induced depression in comparison with the acceptance strategy.

In the present study, we controlled the pre-experimental emotional state across the three groups, indicated by the similar emotion-related measures, such as State/Trait anxiety and depression in different groups. Considering that self-esteem was negatively correlated with depressive responses during stressful situations [65], we also equated the levels of self-esteem across different groups. In addition, there were no significant group differences in the baseline affective ratings and SCR activities. This suggested that the three groups of subjects were similar in the pre-experimental emotional states. Furthermore, the three groups were similar in the habitual use of cognitive reappraisal, expressive suppression and acceptance, ruling out the possibility that these groups had different emotion regulation styles independent of our experimental manipulations. Moreover, our manipulation checks showed that all subjects correctly understood and proficiently used the target strategy during practice trials, and all subjects successfully used the target strategy during the task phase. Therefore, the results should be specific to experimental manipulations of the different emotion regulation instructions.

How to explain the contrasting effects of suppression between Caucasian and Chinese samples? A most likely explanation is that the effect of suppression is culture-specific, as indicated by Butler and colleagues [35]. In contrast to European-American cultures that encourage free emotional expressions, East Asian cultures are characterized by collectivistic cultural norms, which encourage the suppression of emotional displays, in order to avoid hurting others and maintain relational harmony [30]–[34]. The previous studies have showed that culture was an important factor when evaluating the emotional consequences of emotion suppression and expression [85], [86]. In fact, a growing number of literatures have indicated that suppression is associated with less negative emotional experience, better social interactions and more favorable physiological response in individuals with East Asian cultural values [35]–[37]. For instance, Butter and colleagues observed that greater suppression was associated with enhanced negative emotions, increased hostile behaviors or negative interpersonal perceptions in European Americans. By contrast, these adverse outcomes were reduced or reversed in Asian Americans who hold Asian values [35]. Similarly, there was evidence showing that emotional restraint decreased anger experience, anger-expressive behaviors, as well as cardiovascular activity during anger provocation among Asian Americans, but not among European Americans [36]. More recently, Soto [37] observed that suppression was associated with adverse psychological functioning for European Americans, but not for Chinese participants [37]. Consistent with these findings, the suppression strategy produced positive outcomes on both subjective emotional experience and physiological activity (SCR) in the current sample of Chinese participants. However, though Chinese subjects are well known for collectivistic and relational cultural values [33], [34], [85], we need to acknowledge that it was a weakness we did not overtly assess the cultural values of our subjects, which should have then reinforced this explanation.

The present study failed to find positive effects of acceptance on both subjective experience and physiological activity, regardless of whether the task phase or recovery phase was involved. Though many studies observed decreased negative emotion experiences or physiological activity after acceptance instruction [13], [28], [29], there was also evidence showing that acceptance instruction did not produce beneficial outcomes in immediate or long-term emotional measures after watching distressful videos [44], or recalling stressful experiences [87]. A few studies suggested that the effects of emotion regulation strategies might depend on the intensity of the target emotion. For example, Shallcross showed that acceptance predicted lower levels of depressive symptoms after higher, but not lower life stress [22]. These results suggested that the beneficial effects of acceptance in reducing negative affect may be more noticeable at a higher level of emotion intensity [22]. In the present study, we just observed a medium level of depressive emotion induced by our frustration task. This possibly explains why we did not observe beneficial effects of acceptance during regulating depressive emotion. Thus, future studies need to vary the intensity of negative emotion and directly investigate if the regulation effect of acceptance depends on the intensity of target emotion.

One may question that subjects in the suppression group may not suppress emotion displays at late points of emotion generation, as postulated by the process model of emotion regulation [88], but instead subjects might have conducted "repression" continuously in the experiment. In the current study, we used a difficult arithmetic task combined with the feedback manipulation to induce depressive emotion. Specifically, 18 out of 20 trials were manipulated to be associated with negative feedback. Many studies have pointed out that the negative feedback about one's performance is effective in inducing negative, depressive emotional state [53], [56], [57]. For instance, Goodwin and Williams reported that experimental manipulations of success and failure induced depressive and negative affect [56]. In addition, it was reported that the negative feedback was associated with depressive affects in both healthy and depressed individuals [57], [59]. Therefore, the presentation of negative feedbacks, which signals participants' failure in a give trial, should be the critical frustrating event that elicits depressive emotion. Our regulation instruction requires subjects to suppress emotional displays if the frustration happens. Thus, without the presentation of depression-evoking, frustrating event (negative feedback), there is no need for subjects to conduct a suppression process. Also, the triangle-counting task used in this study is rather difficult, to guarantee that subjects' were not highly confident with their answer and consequently, undoubtful about the feedback validity. Therefore, subjects need to focus on the triangle-counting task in the experiment, which implies that there were no enough cognitive resources diverted to other processes if they did not experience frustrating, depressive emotion. Based on these analyses, it was most likely that the suppression group conducted the expressive suppression after they experienced depressive emotion; rather than "repression" continuously through the experiment.

In sum, the present study suggested that, contrary to the conclusions drawn from western samples, the current study using Chinese samples observed that expressive suppression was associated with reduced depressive experiences and smaller physiological activity (SCR) to frustrations compared to the control group. By contrast, the acceptance instruction did not produce beneficial outcomes in these measures during the frustration. These results suggest that cultural background needs to be considered in understanding the emotional consequences of suppression and acceptance.
